# Movement Synchrony in the Psychotherapy of Adolescents With Borderline Personality Pathology – A Dyadic Trait Marker for Resilience?

**DOI:** 10.3389/fpsyg.2021.660516

**Published:** 2021-06-30

**Authors:** Ronan Zimmermann, Lukas Fürer, Johann R. Kleinbub, Fabian T. Ramseyer, Rahel Hütten, Martin Steppan, Klaus Schmeck

**Affiliations:** ^1^Child and Adolescent Psychiatric Research Department, Psychiatric University Hospitals, University of Basel, Basel, Switzerland; ^2^Division of Clinical Psychology and Psychotherapy, Faculty of Psychology, University of Basel, Basel, Switzerland; ^3^Department of Philosophy, Sociology, Education and Applied Psychology, University of Padova, Padova, Italy; ^4^Department of Clinical Psychology and Psychotherapy, Institute of Psychology, University of Bern, Bern, Switzerland; ^5^Division of Personality and Developmental Psychology, Department of Psychology, University of Basel, Basel, Switzerland

**Keywords:** resilience, synchrony, movement, borderline personality disorder, adolescence, psychotherapy, movement synchrony

## Abstract

Movement synchrony describes the coordination of body movements. In psychotherapy, higher movement synchrony between therapist and patient has been associated with higher levels of empathy, therapeutic alliance, better therapy outcome, and fewer drop-outs. The current study investigated movement synchrony during the psychotherapeutic treatment of female adolescents with borderline personality disorder. It was hypothesized that there are higher levels of movement synchrony in the analyzed therapy sessions compared to pseudo-interactions. Further, we tested whether higher levels of movement synchrony correlate with stronger patients’ symptom reduction and whether higher movement synchrony predicts higher post-session ratings. A total of 356 sessions from 16 completed psychotherapies of adolescent patients with BPD were analyzed. Movement synchrony was assessed with motion energy analysis and an index of synchrony was calculated by lagged cross-correlation analysis. As hypothesized, the findings support higher levels of movement synchrony in therapy sessions compared to pseudo-interactions (Cohen’s *d* = 0.85). Additionally, a correlation of movement synchrony with better therapy outcome was found (standardized beta = −0.43 indicating stronger personality functioning impairment reduction). The post-session ratings were negatively associated with higher levels of movement synchrony (standardized beta = −0.1). The relevance of movement synchrony and potential implications for clinical practice are discussed.

## Key Practitioner Message

• In psychotherapy with adolescent patients with borderline personality disorder who completed the therapy, synchrony of the patient’s and the therapist’s movement is associated with better outcome.
• Movement synchrony was negatively correlated with perceived goodness of the session but it was not associated with alliance measured in the middle of the therapy.
• According to theory, alternating cycles of synchrony and independent behavior are required also taking into account that synchrony can take place in different modalities including symbolic levels of abstraction.

## Introduction

Borderline personality disorder (BPD) is a valid and reliable diagnosis in adolescents, and experts call for early detection and early intervention (Chanen et al., 2017; Schmeck et al., 2018). Diagnostic features and stability of symptoms and prevalence rates are similar in adults and adolescents (Kaess et al., 2014; Fonagy et al., 2015). Subthreshold BPD in adolescents is associated with comparable deficits as full-blown BPD in adolescents in terms of psychopathological distress and the impact on health-related quality of life (Kaess et al., 2017). There are several psychotherapeutic approaches specifically developed for adolescent with BPD: e.g., dialectical behavior therapy for adolescents (DBT-A; Fleischhaker et al., 2011), mentalization-based treatment for adolescents (MBT-A; Rossouw and Fonagy, 2012), adolescent identity treatment (AIT; Foelsch et al., 2014), and schema-focused psychotherapy for adolescents (Loose, 2015). The therapeutic process with BPD patients is often challenging because symptoms of BPD (e.g., rejection sensitivity) can hinder the construction of a good therapeutic relationship (McMain et al., 2015) through an increased risk of ruptures of the therapeutic alliance (Schenk et al., 2019, 2020). Psychotherapy process research investigates active ingredients of therapeutic interventions and helps improve therapeutic approaches, as well as informing psychotherapists about the mechanisms of change and how they may leverage these actions (Hardy and Llewelyn, 2015; Zimmermann et al., 2018).

The importance of non verbal relational processes for a positive course of therapy is widely accepted among experts (Flückiger et al., 2018). Most schools of psychotherapy emphasize the relevance of non verbal behavior for the establishment and maintenance of a good therapeutic relationship. Multiple studies report that higher movement synchrony, also called non verbal synchrony, is associated with positive psychotherapy outcome (Ramseyer and Tschacher, 2014, 2016; Altmann et al., 2020). Movement synchrony measures the mutual influence and coordination of non verbal behavior between interaction partners. In the Interpersonal Synchronization Model (In-Sync Model), synchrony has been proposed to embody relationship quality (Koole and Tschacher, 2016) and by this mediation affect outcome. In contrast, Feldman’s resilience model depicts synchrony as the core mechanism in building resilience (Feldman, 2020).

The current study investigates movement synchrony in the psychotherapy of adolescents with borderline personality disorder. The assessment of movement synchrony could provide further insight into the quality of non verbal interactions between BPD youth and psychotherapists.

### Hypothesis 1: Significant Levels of Movement Synchrony Are Observable in Psychotherapy Sessions With Adolescents With BPD

The study investigates the question whether in the psychotherapy of adolescents with Borderline Personality Pathology, the therapist and the patient show movement synchrony above a level that would be expected by coincidence. The distinction between measured movement synchrony and synchrony that would be expected by chance is important, as it confirms that movement synchrony exists and can be measured in the addressed context. While significant movement synchrony was shown in clinical interviews with adult patients with BPD (Ramseyer et al., 2020), the psychotherapeutic communication between adolescent patients and adult psychotherapists might differ from that in adults (Zimmermann et al., 2020). For instance, in adolescents at risk for anxiety, movement synchrony was absent when measured in a setting with the youths’ fathers (Roman-Juan et al., 2020). Similarly, synchrony might be absent in young patients with BPD and their psychotherapist. Despite this possibility, movement synchrony above chance level was expected to be present in this setting.

### Hypothesis 2: Higher Levels of Movement Synchrony Are Associated With Better Therapy Outcome

Synchrony is expected to correlate with the outcome of psychotherapy. Outcome was assessed in terms of psychosocial functioning (CGAS; Shaffer et al., 1983), and personality functioning (Goth et al., 2018).

### Hypothesis 3: Higher Levels of Movement Synchrony Are Associated With Better Post-Session Evaluations Rated by Patient and Therapist

Studies using MEA often analyzed time-limited parts of single therapy sessions (Schoenherr et al., 2019a) or investigated only a subset of sessions (Ramseyer and Tschacher, 2011; Paulick et al., 2018a). These sampling strategies increase the risk of missing important aspects of the dynamic process inherent in movement synchrony. Despite the use of different sampling strategies, movement synchrony was robustly correlated with the outcome of psychotherapy, suggesting that movement synchrony is a trait-like dyadic phenomenon (Zilcha-Mano and Ramseyer, 2020). However, two recent studies that addressed this distinction, showed that only the state-like aspect of movement synchrony was positively associated with alliance (Prinz et al., 2020; Cohen et al., 2021). In the current study, we were interested in the variability of movement synchrony within the dyad and whether there is a trend observable across the course of therapy. We expected that movement synchrony is relatively characteristic for a given dyad (trait-like effect). Furthermore, we expected an impact of synchrony in a given session on the perceived quality of the session (state-like effect).

## Materials and Methods

### Design

The study is part of the multicenter study “Evaluation of Adolescent Identity Treatment” (Schmeck et al., 2018; Zimmermann et al., 2018) that has been registered at clinicaltrials.gov (NCT02518906). Current analyses are based on the data collected at one participating center (Psychiatric Hospitals of the University of Basel using AIT). Ethical approval was obtained from the local ethics committee. All adolescents, their parents, and the therapists provided written informed consent for participation.

### Sample

The following inclusion criteria were applied for the patients: age 13–19 years; ≥3 BPD criteria according to the Structured Clinical Interview for Diagnostic and Statistical Manual of Mental Disorders, Fourth Edition, Axis II Personality Disorders (First et al., 1997), and identity diffusion according to the Assessment of Identity Development in Adolescence (total *t*-score ≥ 60; Goth et al., 2012). Applied exclusion criteria were IQ < 80, psychotic disorders, pervasive developmental disorders, severe somatic or neurological disorders, severe and persistent substance addiction, antisocial personality disorder and necessity for inpatient treatment (for details, see Zimmermann et al., 2018).

In the current study, data of the 16 completed psychotherapies was used for which psychotherapy outcome was available. The included psychotherapies needed to include at least half of the planned 25 psychotherapy sessions and, additionally, outcome data needed to be available. The sample originally comprised 16 female and one male patient. The male patient was excluded from the sample to make the sample more homogeneous. Mean age of the patients was 16.6 (SD = 1.5).

In total, 381 sessions were administered in the sample. Fourteen sessions were not available in one patient because of withdrawal of consent. Another 11 sessions were missing in different psychotherapies due to technical or human failure (e.g., recording not started or video not saved). The remaining 356 sessions were available for analysis. See Supplementary Figure 1 for a representation of the available data.

### Treatment – Adolescent Identity Treatment (AIT)

Adolescent identity treatment (Foelsch et al., 2014) is a treatment for adolescents with personality disorders (PD). AIT integrates modified elements of transference-focused psychotherapy (TFP) (Clarkin et al., 1999) with psychoeducation, home plans and systemic work with parents and institutions. The main techniques of AIT are clarification, confrontation, and interpretation. Therapists focus on the affects in the here and now and on dominant object relationship dyads. All AIT therapists had advanced training in AIT and came from medicine or psychology backgrounds. Therapists were supervised on a weekly basis. Adherence and competence of the psychotherapist was evaluated by one of the authors of the AIT manual.

### Assessments

All instruments and their time of administration used in this study are described in detail elsewhere (Zimmermann et al., 2018). The following section will focus on the instruments relevant for the current analyses.

Motion energy was automatically measured in video-recordings of therapy sessions. Session impact was measured with the Session Evaluation Questionnaire (SEQ; Stiles, 1980) after each session. The outcome measures Levels of Personality Functioning Questionnaire (LoPF-Q 12–18; Goth et al., 2018) and Children Global Assessment Scale (CGAS; Shaffer et al., 1983) were measured at baseline and at 1 year after the baseline (1YFU). For outcome, the difference between baseline and the 1YFU was used. For the Levels of Personality Functioning Questionnaire the outcome score was inverted so that high scores signify a better therapeutic outcome.

The LoPF-Q 12–18 consists of 97 5-point Likert-type items and was constructed specifically for adolescent populations. The LoPF model encompasses four dimensions, two of which are self-related (Identity and Self-Direction) and two of which are of an interpersonal nature (Empathy and Intimacy). The dimensions have a pathological and healthy pole and can be aggregated to a total score that represents the severity of personality pathology in terms of personality functioning, with high values indicating severe personality pathology. The total score differentiated those adolescents with personality disorders from a school sample with a large effect size (Cohen’s *d* = 2.3). Moreover, the test showed good scale reliability with a Cronbach’s Alpha of 0.97 for the global scale (Goth et al., 2018).

Children Global Assessment Scale is a clinician-rated measurement to assess overall level of functioning in children (Shaffer et al., 1983). The CGAS score indicates how well the level of functioning is developed in a number of aspects related to the psychological and social functioning of a child in different environments (i.e., at home, at school, and with peers). It is rated on a scale ranging from 1 to 100. A higher score represents a better functioning. According to the CGAS score children can be assigned to ten different categories ranging from “extremely impaired” to “doing very well.” The authors report an inter-rater reliability of 0.84, a test-retest reliability of 0.85 and evidence of discriminative validity (Shaffer et al., 1983; see also Bird et al., 1993).

The SEQ was filled out by the therapist and the patient immediately after each psychotherapy session. The instructions for this read as follows: “Please circle the appropriate number to show how you feel about this session.” The items are answered on a seven-point Likert-type scale. Goodness of the session was evaluated by means of one item that asked the participants to rate the session from bad to good. The SEQ has demonstrated good content, criterion, and construct validity (Stiles, 1980; Stiles and Snow, 1984; Stiles et al., 1990).

### Motion Energy Analysis (MEA)

Two frontally wall-mounted cameras video-recorded patient and therapist separately. The recordings were cut to include only the actual psychotherapeutic process from the moment the therapist invited the patient to start the session in a sitting position to the moment the therapist terminated the session. The timestamps of the videos were synchronized at a millisecond level.

Motion energy analysis works with frame-by-frame pixel changes to measure the movement of the protagonists in predefined regions of interest (ROIs). The recordings were converted to grayscale. MEA computes the differences in these grayscale pictures for all consecutive pictures defined as motion energy by Grammer et al. (1999) and adapted to the psychotherapy setting by Ramseyer and Tschacher (2011). The patient and therapist videos were analyzed with the MATLAB based method of MEA made available by Altmann and Schoenherr (Altmann et al., 2020; see https://github.com/10101-00001/MEA/ for downloading the software).

**FIGURE 1:**
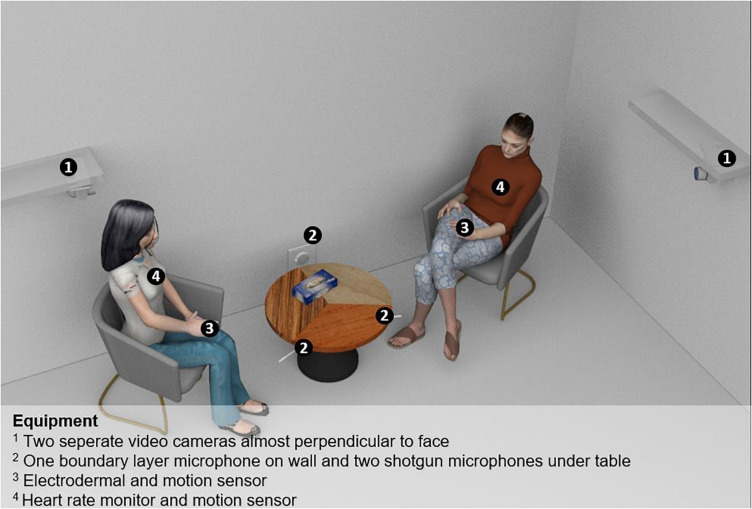
Study setting. 1, Two separate video cameras almost perpendicular to face; 2, One boundary layer microphone on wall and two shotgun microphones under table; 3, Electrodermal and motion sensor; 4, Heart rate motion sensor.

Slight modifications were made to run Altmann and Schoenherr’s method on separate patient and therapist videos and to use the same ROIs for all patient and all therapist videos, respectively. This was possible due to the stationary camera setting (see Figure 1) and it allowed for batch processing of the videos. We used one ROI per person that covered body movement including the head. No separate ROI was used for the head. ROIs for the patient and therapist videos were defined based on a manual review of the moving range of the participants in all videos. The “threshold” setting of Altmann’s and Schoenherr’s MEA method was set to 12, which is conservative (see manual of Altmann and Schoenherr on github). The range of the motion energy time series was standardized for ROI size using Altmann’s and Schoenherr’s pre-processing. To correct video errors, MEA values ≥10 SD, were excluded and then replaced by carrying the last value forward. Additionally, values were replaced when motion energy in the control ROIs was ≥5. Finally, a butterworth filter was used to smooth the results of the MEA.

### Quantification of Movement Synchrony

Further steps were calculated with the R package rMEA by Kleinbub and Ramseyer (2021). The pre-processed motion energy time-series were used to compute cross-correlations as an indicator of the level of synchrony between patient and therapist: the time-series of patient and therapist were cross-correlated within window segments of a 1-min duration without overlap. For each window, cross-correlations for negative and positive time-lags up to 5 s were computed (total of 50 steps of 0.1 s in each direction). Finally, these cross-correlations were aggregated to a grand-average, a single numeric value per session of all cross-correlations. These parameters follow previously used settings established in research on movement synchrony in psychotherapy (Ramseyer and Tschacher, 2011). For outcome analyses, a global value of the amount of movement synchrony was generated by calculating the median for each dyad across all session-level grand-averages.

### Statistical Analyses

All statistical analyses were performed by the software R (R Core Team, 2020). A level of *p* < 0.05 was used for the assessment of statistical significance. *P*-values were adjusted for multiple hypotheses testing using Benjamini’s and Hochberg’s method (Jafari and Ansari-Pour, 2019).

#### Hypothesis 1: Significant Levels of Movement Synchrony Are Observable in Psychotherapy Sessions With Adolescents

Presence of synchrony was tested against a pseudo-synchrony data set (*n* = 500) created by between-subjects shuffling of all motion energy time-series of the original data set (see Kleinbub and Ramseyer, 2021 for details about the procedure). A Welch Two Sample *t*-test was applied to verify that genuine synchrony is significantly above pseudo-synchrony. Additionally, Cohen’s *d* effect sizes were calculated.

#### Hypothesis 2: Higher Levels of Movement Synchrony Are Associated With Better Therapy Outcome (Personality Functioning and Psychosocial Functioning) and Hypothesis 3: Higher Levels of Movement Synchrony Are Associated With Better Patient and Therapist Post Session Evaluations (SEQ)

Hypothesis 2 and 3 were tested in the same linear random mixed effect model (Bates et al., 2015; Kuznetsova et al., 2017). Intercept by dyad was used as random effect. The grand mean of movement synchrony per session standardized by subtracting the mean of the pseudo-synchrony data and dividing by its standard deviation was used as dependent variable. Synchrony was chosen as dependent variable to be able to test the hypothesized correlations along with the potentially confounding variables and to, additionally, check for shared variance of the predictors. The following hypothesis related predictors were used in the model:

– Hypothesis 2: Change in LoPF-Q 12–18 total score between baseline and therapy follow-up (smaller numbers indicating better outcome).
– Hypothesis 2: Change in CGAS score (greater numbers indicate better outcome).
– Hypothesis 3: Average of the therapist and the patient goodness ratings (SEQ).

#### Further Potentially Relevant Variables (Confounders)

Session number was used to account for trends over the psychotherapy. Alliance at session 12 (mid-therapy), measured with the overall score of the Working Alliance Inventory-Short Revised (WAI-SR) (Munder et al., 2010), was used because it is hypothesized to be relevant according to the In-Sync model. Finally, the baseline scores of the outcome parameters were used to control for an effect of psychopathology.

A stepwise elimination procedure (Kuznetsova et al., 2017) was used to select significant predictors. For the resulting model, standardized coefficients and standard errors are reported. Additionally, the marginal and conditional coefficients of determination are reported (Nakagawa and Schielzeth, 2013).

## Results

### Hypothesis 1: Significant Levels of Movement Synchrony Are Observable in Psychotherapy Sessions With Adolescents

Presence of synchrony in the original data set vs. pseudo-synchrony was confirmed (*p* < 0.01) with a high effect size (Cohen’s *d* = 0.85).

### Hypothesis 2 and Hypothesis 3

Outcome measured as CGAS as well as the baseline measurement of the LoPF-Q 12–18 and the CGAS were dropped from the model by the stepwise elimination procedure, meaning that these variables were not significant predictors for movement synchrony. Alliance was also dropped by this procedure. The random intercept by dyad was significant and remained in the model according to the stepwise procedure (Supplementary Table 1 shows the details of the stepwise elimination procedure, Supplementary Table 2 shows the correlation of synchrony with all predictors in the model without the elimination of non-significant predictors).

Fixed effects of the random mixed effects model are shown in Table 1. Outcome measured as change in total score of the LoPF-Q 12–18 shows a significant correlation with movement synchrony: therapies with more synchrony show more improvement in personality functioning.

**TABLE 1:** Fixed effects and random effects model for hypothesis 2 and hypothesis 3.

	Synchrony							
Predictors	Estimates	Std. error	Std. beta	Standardized std. error	CI	Standardized CI	*p*	Adjusted *p*
(Intercept)	1.41	0.43	−0.04	0.18	0.56–2.26	−0.39–0.31	0.001	
Outcome (change in LoPF)	−0.01	0.00	−0.43	0.18	−0.02 – −0.00	−0.78– −0.09	0.014	0.040
Goodness of session (SEQ)	−0.14	0.06	−0.10	0.04	−0.26 – −0.03	−0.18 – −0.02	0.012	0.024
Session number	−0.02	0.01	−0.08	0.04	−0.03 – −0.00	−0.16 – −0.01	0.032	
**Random effects**								
σ^2^		0.71						
τ_00 id_		0.67						
ICC		0.48						
Marginal *R*^2^/Conditional *R*^2^		0.180/0.577						
Deviance		803.340						

*The table shows the predictors of synchrony that were selected by the stepwise elimination procedure. Adjusted p-values are shown for variables used in the three hypotheses of the article.*

Regarding the predictor goodness (SEQ), the model showed a significant correlation in the sense that, unexpectedly, session quality was rated lower, when more synchrony was measured in a session.

Session number was also significantly correlated with synchrony in the sessions, showing a decrease of synchrony over the sessions.

**FIGURE 2:**
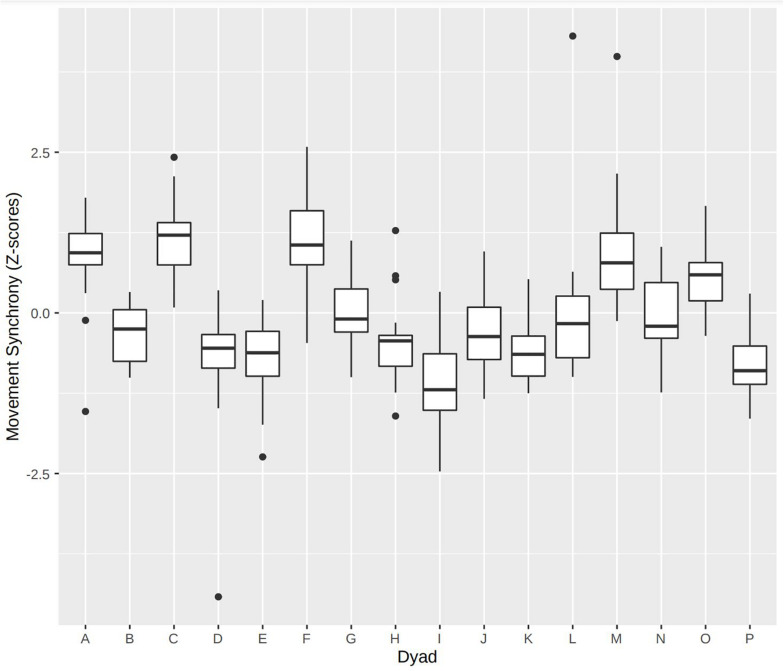
Variability of movement synchrony between dyads. Movement synchrony is characteristic for the dyads.

The marginal coefficient of determination for the overall model was 0.18 indicating that a considerable part of variability in synchrony was explained by the finally selected fixed effects. The conditional coefficient of determination (which combines the random and fixed effects) was 0.58 (Nakagawa and Schielzeth, 2013) showing that variability in synchrony is mostly explained by the dyad which was used a grouping variable for the random effect. Figure 2 confirms the impression; variability of synchrony within the dyads is small compared to the between-dyad spectrum.

## Discussion

The study confirmed that significant movement synchrony is present in Adolescent Identity Treatment of youth with borderline personality disorder (Hypothesis 1). With an effect size of *d* = 0.85, this effect was large in the current sample, a result also found in adult psychotherapy (*d* = 0.6; Ramseyer and Tschacher, 2011). Additionally, the study confirms that higher movement synchrony in the patient therapist dyad is correlated to better outcome regarding self-rated personality functioning but is surprisingly not correlated with psychosocial functioning as rated by a clinician (Hypothesis 2). Interestingly, movement synchrony was not correlated to the initial level of personality functioning but only to the change from baseline to follow-up. The results can be interpreted in the sense that the temporal coordination in the dyad is of importance for the effect of psychotherapy. Further, we did not confirm that sessions with more synchrony are rated better by patient and therapist (Hypothesis 3). This would have been in line with the result regarding the overall outcome of the psychotherapy. However, unexpectedly, sessions with more movement synchrony were rated as being less good. A similar finding was reported in an idiographically oriented sample of adult psychotherapy: Sessions characterized by little progress (from the therapist’s perspective) were marked by high synchrony (Ramseyer, 2020).

Regarding the investigated confounders, we found a decrease of synchrony over the sessions but we did not find a correlation of synchrony with working alliance. The latter finding does not fit well with the In-Sync model (Koole and Tschacher, 2016), which assumes a connection of synchrony with alliance and by this route a subsequent effect on outcome. Schoenherr et al. (2019b) concluded that there might be different facets or constructs of synchrony and that they might differ in how they relate to other constructs. Paulick et al. (2018b) found evidence that there are diagnosis specific patterns in movement synchrony. Thus, it is possible that the role of synchrony in psychotherapy treatment of adolescent with personality disorders deviates from its role in adult samples. Adolescence is recognized as a critical and sensitive period for personal development and maturation. It involves increased neuroplasticity and it is thought to present a window of opportunity for neuropsychiatric interventions (Marín, 2016). With its neurodevelopmental approach, Feldman’s resilience model might shed light on some of the observed results. The model describes that external regulation of the infant’s immature brain by the mature brain (e.g., the mother) allows to fine-tune the neurobiological and behavioral systems to life within the social ecology. Feldman (2020, p. 133) argues that “the tuning of the infant’s brain to life within the ecological niche and its distinct hardships marks the very essence of resilience and that processes that participate in such tuning define what resilience “is,” and should become the focus in resilience theory and research.” Biobehavioral synchrony defined as “the coordination of biological and behavioral signals between social partners during moments of social contact” (Feldman, 2020, p. 133) forms a secure basis in the dyadic relationship which is the platform for expanding symbolic and interpersonal complexity. Finally, the increased diversity of functioning amidst a core order is what defines resilience according to this model.

Resilience is defined as a positive outcome despite adversity (Feldman, 2020). While resilience was not explicitly measured in the current study, it is possible to assume a link between the outcome of a psychotherapy and achieving to build or activate resilience during psychotherapy. In our view, better resilience could potentially translate to more adaptive personality functioning.

To reintroduce some complexity, it needs to be mentioned that synchrony should not be considered as a continual mode of operation. Rather, alternation of synchronous and non-synchronous behavioral modes should be expected with the greater portion of time spent in mismatch (Tronick, 1989; Boker and Rotondo, 2002; Feldman, 2020). These match-mismatch cycles allow for building autonomy and lastly resilience according to Feldman’s model (Feldman, 2020). A phenomenon of hyper-synchrony can sometimes be observed which can then be characterized as a rigid pattern and is associated with regulatory difficulties (Feldman, 2020). While synchrony is an essential mechanism, it needs to play its role in a dynamic and flexible system to result in resilience. In this framework, our result of less good session ratings related to more synchrony might make sense and could potentially reflect a negatively perceived lack of flexibility and autonomy. More autonomy of the patient toward the end of the psychotherapy might also be a potential explanation for the observed trend to less synchrony with higher session number (Krause et al., 2007).

In the published study protocol of the current study, we had formulated the *a priori* hypothesis “that interpersonal synchrony might be a basic mechanism of psychotherapy in which patient and therapist have to create a shared psychotherapeutic space.” (Zimmermann et al., 2018, p. 188). Our study cannot be a strong scientific argument for the validity of Feldman’s model in psychotherapy because of the small sample size and the lack of a clear methodology to measure resilience. However, future psychotherapy research should take this model into consideration especially related to the study of interpersonal synchrony in psychotherapy. The comparison and, potentially, the synthesis of different theoretical models in light of the available data might lead to a better understanding of interpersonal synchrony in the context of psychotherapy.

As a sidenote, and to put previous findings in this sample into perspective, in a previous study on silence in psychotherapy, we showed that in the present sample, silence can be problematic for the psychotherapeutic process (Zimmermann et al., 2020). A further study based on the same data showed that there is an increased risk of withdrawal ruptures (Schenk et al., 2019, 2020) during psychotherapy in youth with BPD. We believe that these phenomena (silence and ruptures) are part of the normal psychotherapeutic process but that they imply a risk of being detrimental to the psychotherapeutic process because they can rigidify the interaction and hinder synchronization of the dyad in a larger sense.

As a major limitation, movement synchrony represents a rather basic method for measuring synchrony and it does not account for the more abstract and symbolic ways in which adolescent and adult individuals may synchronize. This also is a theoretical gap for using Feldman’s model, as the model is based on infant research. Still, a number of studies have shown that the phenomenon of movement synchrony can be scientifically described with robust effects on outcome (Ramseyer and Tschacher, 2011; Altmann et al., 2020). Again, this shows the status of movement synchrony as a fundamental mechanism in psychotherapy. An additional limitation is the small sample size. Therefore, the authors recommend a careful consideration of the results until they can be replicated. However, similar results were shown in larger samples and with other study populations which might support the validity of the current analyses. It would be of interest to directly compare synchrony in patients with and without borderline personality disorders to get more insights on the meaning of synchrony in psychotherapy. A further recommendation would be to look at significant therapeutic events to get a clearer picture of the circumstances under which synchrony can have a certain function.

The number of investigated dyads in this study is lower than planned in the study protocol (Zimmermann et al., 2018), because we only used data from one of the participating centers (see section “Materials and Methods”). Quality of the video recordings at the second center was less standardized and difficult to compare to the recordings used in the current study (different angle, one camera instead of two, and, in some videos, problematic lighting conditions). Additionally, the study recruited only 23 of the planned 29 cases and excluded male patients for better homogeneity of the sample. A high dropout rate was expected and the current study only investigated completed psychotherapies as post therapeutic measures were not available for dropout cases.

As an improvement compared to previous literature, the current study has employed a fully automatic method for motion energy analysis which has allowed the study of full psychotherapies in terms of using the full length of the sessions and the total number of sessions. This allowed us to test for trends in movement synchrony (e.g., an increase of movement synchrony over the psychotherapy) as well as to consider the variance of movement synchrony in and between the dyads.

## Data Availability Statement

The data supporting the conclusions of this article will be made available by the authors, on reasonable request without undue reservation.

## Ethics Statement

This study involving human participants was reviewed and approved by the Ethikkommission Nordwest- und Zentralschweiz (EKNZ). Written informed consent to participate in this study was provided by the participants and a legal guardian in case the participants were not of legal age.

## Author Contributions

All authors listed have made a substantial, direct and intellectual contribution to the work, and approved it for publication.

## Conflict of Interest

The authors declare that the research was conducted in the absence of any commercial or financial relationships that could be construed as a potential conflict of interest.
