# Supplementary figures and images for: Movement Synchrony in the Psychotherapy of Adolescents With Borderline Personality Pathology – A Dyadic Trait Marker for Resilience?
Source: Front Psychol. 2021 Jun 30;12:660516. doi: 10.3389/fpsyg.2021.660516 (PMC8277930; doi:10.3389/fpsyg.2021.660516)

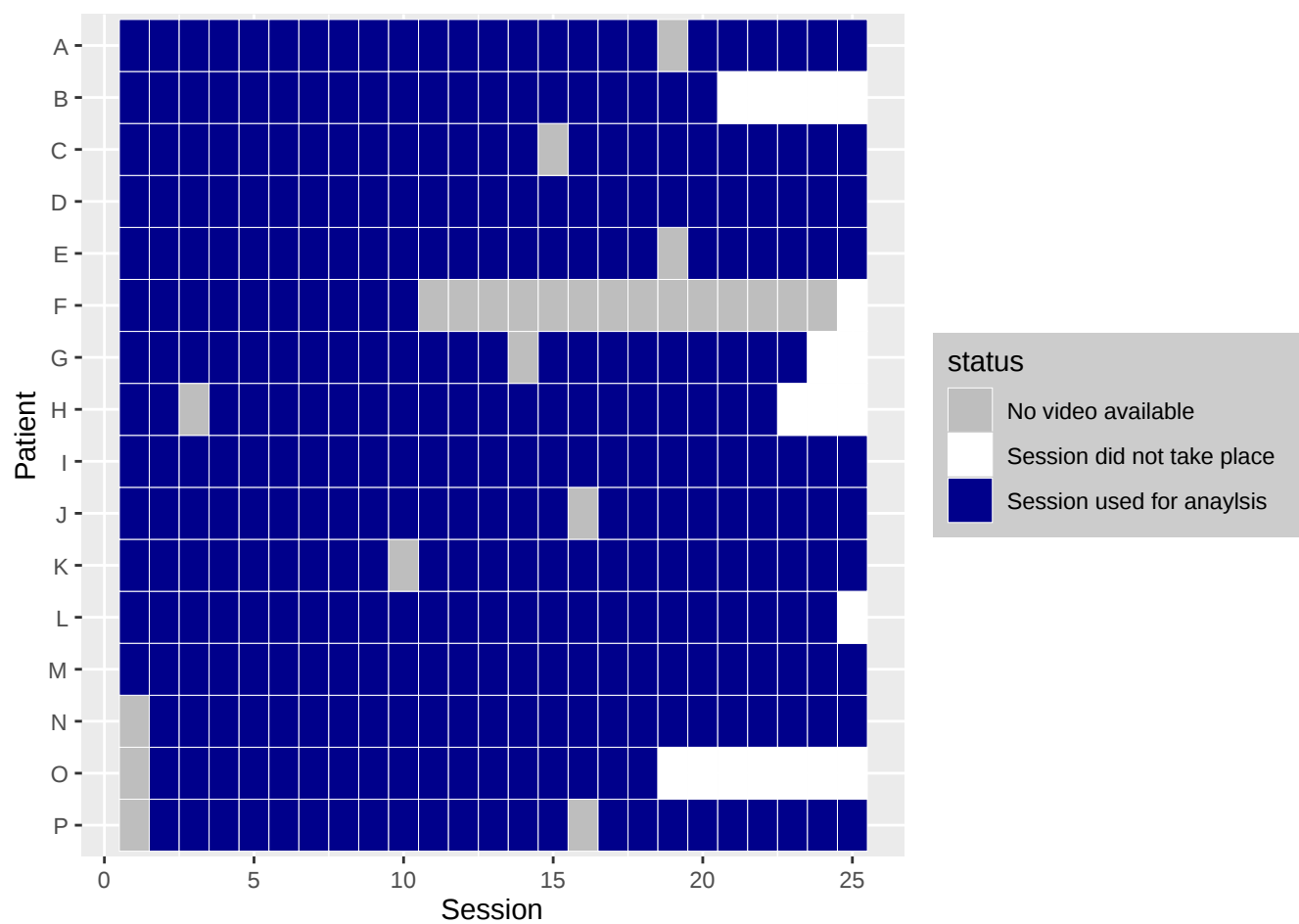

Supplement: Supplementary file 1 [file Data_Sheet_1.PDF]
