# Supplementary material for: Movement Synchrony in the Psychotherapy of Adolescents With Borderline Personality Pathology – A Dyadic Trait Marker for Resilience?
Source: Front Psychol. 2021 Jun 30;12:660516. doi: 10.3389/fpsyg.2021.660516 (PMC8277930; doi:10.3389/fpsyg.2021.660516)
Supplement: Supplementary file 2 [file Table_1.DOCX]

**Table A - Details of stepwise backwards elimination procedure**

| Random effects |  | Eliminated | npar | logLik | AIC | LRT | Df | p-value | |
| --- | --- | --- | --- | --- | --- | --- | --- | --- | --- |
|  |  |  | 10 | -420.8 | 861.6 |  |  |  |  |
|  | (1 \| id) | 0 | 9 | -491.2 | 1000.5 | 140.9 | 1 | <0.0001 | *** |
| Fixed effects |  | Eliminated | Sum Sq | Mean Sq | Num DF | Den DF | F value | p-value | |
|  | wai | 1 | 0.001 | 0.001 | 1 | 8.03 | 0.002 | 0.9688 |  |
|  | lopf_tot | 2 | 0.007 | 0.007 | 1 | 9.02 | 0.01 | 0.9207 |  |
|  | cgas_out | 3 | 0.039 | 0.039 | 1 | 10.3 | 0.055 | 0.8186 |  |
|  | cgas | 4 | 0.749 | 0.749 | 1 | 10.92 | 1.051 | 0.3275 |  |
|  | lopf_tot_out | 0 | 4.276 | 4.276 | 1 | 12.1 | 6.001 | 0.0305 | * |
|  | good | 0 | 4.546 | 4.546 | 1 | 292.85 | 6.38 | 0.0121 | * |
|  | session | 0 | 3.28 | 3.28 | 1 | 292.21 | 4.603 | 0.0327 | * |

*Note:* Backward reduced random- and fixed-effect table. Model found: sync ~ lopf_tot_out + good + session + (1 | id); Degrees of freedom method: Satterthwaite; Significance codes: *** p < 0.001; ** p < 0.01; * p < 0.05. Abbreviations: *‘(1|id)’,* random intercept for each participant; *‘wai’,* Working Alliance Inventory; *‘lopf_tot’,* Levels of Personality Functioning Questionnaire (LoPF-Q 12-18) total score at baseline; *‘lopf_tot_out’,* change in LoPF-Q 12-18 total scores; *‘cgas’,* Children Global Assessment Scale (CGAS) score at baseline; *‘cgas_out’,* change in CGAS scores; *‘good’,* goodness of sessions assessed through the Session Evaluation Questionnaire (SEQ); *‘session’,* the session number.
