# Supplementary material for: Movement Synchrony in the Psychotherapy of Adolescents With Borderline Personality Pathology – A Dyadic Trait Marker for Resilience?
Source: Front Psychol. 2021 Jun 30;12:660516. doi: 10.3389/fpsyg.2021.660516 (PMC8277930; doi:10.3389/fpsyg.2021.660516)
Supplement: Supplementary file 3 [file Table_2.DOCX]

**Table B - Fixed effects of random effects model for hypothesis 2 and hypothesis 3 showing all predictors including those removed by stepwise elimination procedure.**

| **Synchrony** | | | | | |  |
| --- | --- | --- | --- | --- | --- | --- |
| *Predictors* | *Estimates* | *std. Error* | *std. Beta* | *standardized std. Error* | *CI* | *standardized CI* |
| (Intercept) | -0.03 | 5.35 | -0.04 | 0.21 | -10.5 – 10.4 | -0.45 – 0.37 |
| Outcome (change in LoPF) | -0.01 | 0.01 | -0.44 | 0.25 | -0.02 – 0.00 | -0.94 – 0.06 |
| Outcome (change in cgas) | -0.01 | 0.03 | -0.06 | 0.24 | -0.07 – 0.06 | -0.52 – 0.41 |
| CGAS baseline | 0.04 | 0.06 | 0.17 | 0.25 | -0.08 – 0.15 | -0.32 – 0.66 |
| LoPF baseline | -0.00 | 0.01 | -0.03 | 0.30 | -0.02 – 0.02 | -0.61 – 0.55 |
| Goodness of session (SEQ) | -0.15 | 0.06 | -0.11 | 0.04 | -0.26 – -0.03 | -0.19 – -0.02 |
| Session number | -0.02 | 0.01 | -0.09 | 0.04 | -0.03 – -0.00 | -0.16 – -0.01 |
| Working Alliance (12^th^ session) | -0.02 | 0.52 | -0.01 | 0.27 | -1.04 – 1.00 | -0.53 – 0.51 |
| **Random Effects** | | | | | | |
| σ^2^ | 0.71 | | | | | |
| τ_00_ _id_ | 0.92 | | | | | |
| ICC | 0.56 | | | | | |
| Marginal R^2^ / Conditional R^2^ | 0.182 / 0.643 | | | | | |
| Deviance | 803.556 | | | | | |
